# Robot-assisted thoracic surgery versus video-assisted thoracic surgery for lung lobectomy or segmentectomy in patients with non-small cell lung cancer: a meta-analysis

**DOI:** 10.1186/s12885-021-08241-5

**Published:** 2021-05-03

**Authors:** Jianglei Ma, Xiaoyao Li, Shifu Zhao, Jiawei Wang, Wujia Zhang, Guangyuan Sun

**Affiliations:** 1 Student of the College of Basic Medical Sciences, Naval Medical University, No. 800 Xiangyin Road, Yangpu District, Shanghai, 200433 China; 2 Department of Thoracic Surgery, Changzheng Hospital, Naval Medical University, No. 415 Fengyang Road, Huangpu District, Shanghai, 200003 China

**Keywords:** Robot-assisted thoracic surgery, Video-assisted thoracic surgery, Lobectomy, Segmentectomy, Non-small cell lung cancer

## Abstract

**Background:**

It remains no clear conclusion about which is better between robot-assisted thoracic surgery (RATS) and video-assisted thoracic surgery (VATS) for the treatment of patients with non-small cell lung cancer (NSCLC). Therefore, this meta-analysis aimed to compare the short-term and long-term efficacy between RATS and VATS for NSCLC.

**Methods:**

Pubmed, Cochrane Library, Embase, China National Knowledge Infrastructure (CNKI), Medline, and Web of Science databases were comprehensively searched for studies published before December 2020. The quality of the articles was evaluated using the Newcastle-Ottawa Scale (NOS) and the data analyzed using the Review Manager 5.3 software. Fixed or random effect models were applied according to heterogeneity. Subgroup analysis and sensitivity analysis were conducted.

**Results:**

A total of 18 studies including 11,247 patients were included in the meta-analyses, of which 5114 patients were in the RATS group and 6133 in the VATS group. Compared with VATS, RATS was associated with less blood loss (WMD = − 50.40, 95% CI -90.32 ~ − 10.48, *P* = 0.010), lower conversion rate (OR = 0.50, 95% CI 0.43 ~ 0.60, *P* < 0.001), more harvested lymph nodes (WMD = 1.72, 95% CI 0.63 ~ 2.81, *P* = 0.002) and stations (WMD = 0.51, 95% CI 0.15 ~ 0.86, *P* = 0.005), shorter duration of postoperative chest tube drainage (WMD = − 0.61, 95% CI -0.78 ~ − 0.44, *P* < 0.001) and hospital stay (WMD = − 1.12, 95% CI -1.58 ~ − 0.66, *P* < 0.001), lower overall complication rate (OR = 0.90, 95% CI 0.83 ~ 0.99, *P* = 0.020), lower recurrence rate (OR = 0.51, 95% CI 0.36 ~ 0.72, *P* < 0.001), and higher cost (WMD = 3909.87 USD, 95% CI 3706.90 ~ 4112.84, *P <* 0.001). There was no significant difference between RATS and VATS in operative time, mortality, overall survival (OS), and disease-free survival (DFS). Sensitivity analysis showed that no significant differences were found between the two techniques in conversion rate, number of harvested lymph nodes and stations, and overall complication.

**Conclusions:**

The results revealed that RATS is a feasible and safe technique compared with VATS in terms of short-term and long-term outcomes. Moreover, more randomized controlled trials comparing the two techniques with rigorous study designs are still essential to evaluate the value of robotic surgery for NSCLC.

**Supplementary Information:**

The online version contains supplementary material available at 10.1186/s12885-021-08241-5.

## Introduction

At present, lung cancer is still the leading cause of cancer morbidity and mortality worldwide, with NSCLC accounting for 80–85% of all lung cancer cases [1, 2]. Surgical resection, including traditional thoracotomy and minimally invasive surgery (MIS), is still the current preferred treatment for lung cancer, and surgical resection with lymphadenectomy is considered to be the standard treatment for patients with NSCLC at an early stage [3, 4]. Since the initial VATS lobectomy was first performed in the early 1990s, VATS has been widely used in thoracic surgery all over the world, and its safety and effectiveness in the treatment of early NSCLC have been confirmed, which makes it become the main surgical procedure for lung cancer resection [5–8].

In recent years, VATS has been recognized for its advantages of MIS in the treatment of NSCLC, such as less blood loss, less postoperative pain, fewer complications, faster recovery, shorter hospital stay, and lower mortality rates [9, 10]. Clinical trials comparing VATS with conventional thoracotomy have shown that VATS has superior perioperative outcomes and improved long-term survival [11, 12]. Although VATS has such advantages, it has also limitations of itself, including two-dimensional vision, difficult hand-eye coordination, amplification of hand tremor, steep learning curve, lack of flexibility and limited ranges of instrument movement, which may restrict the development of this technique [13, 14].

Advantages of RATS include high definition three-dimensional stereo video, improved ergonomics less steep learning curve, tremor suppression and better maneuverability of instruments, which can promote complex movements in a closed space and influence the perioperative outcomes [15–17]. Da Vinci robot, currently the latest generation of robotic surgery systems in the world, has unlocked a new era of MIS and been widely used in urinary tract, cardiovascular, hepatobiliary, gastrointestinal and gynecological surgery [18]. Since Melfi et al. [19] firstly applied it to lung surgery in 2002, studies on RATS have been widely reported.

Many types of research published in the past 10 years have confirmed the feasibility and safety of RATS, which are meaningful in highlighting the status of RATS in the treatment of NSCLC [20–22]. However, these researches included single-center studies with the small sample size and different appraise systems of complications, which limited them to conclude objective results. Moreover, RATS may be restricted by its higher hospital costs and longer operative time. Therefore, there is no clear conclusion whether RATS can achieve an equal or even better surgical effect when compared with VATS. We conducted this meta-analysis to explore and compare the short-term and long-term outcomes of RATS versus VATS for lung lobectomy or segmentectomy in patients with NSCLC.

## Methods

### Search strategy

The Preferred Reporting Items for Systematic Reviews and Meta-Analyses (PRISMA) statement was applied to perform this study and the PRISMA checklist was completed [23]. A systematic literature search was performed in Pubmed, Cochrane Library, Embase, CNKI, Medline, and Web of Science for studies published before December 2020 that assessed the comparison between RATS and VATS in the treatment of NSCLC, using the following searching terms: “robot-assisted OR robot-assisted thoracic surgery OR robot OR robotic OR computer-assisted surgery OR da Vinci”, “video-assisted OR video-assisted thoracic surgery OR video OR thoracoscopic”, “lung lobectomy OR segmentectomy”, and “non-small cell lung cancer OR lung cancer OR lung carcinoma”. In addition, the reference lists of all relevant articles were also searched to identify the additional relevant literature. There was no restriction on the language of the articles.

### Inclusion criteria and exclusion criteria

The included studies must meet the following criteria: (1) Clinical studies comparing RATS with VATS for lung lobectomy or segmentectomy in patients with NSCLC; (2) Full-text articles that reported necessary data for statistical analysis, including at least one of the following outcomes: operative time, estimated blood loss, conversion, the number of dissected lymph nodes, length of hospital stay, postoperative duration of drainage, postoperative complications, mortality, OS, DFS, recurrence rate, and cost; (3) If the same team or institution reported more than one studies, the latest, the larger scale number or high-quality publication were included. If two or more studies contained totally different cases from the same center, we still analyzed the data from these studies.

Articles were excluded if they met any of the following criteria: (1) Case reports, review articles, animal experimental studies, letters to the editor, meeting abstracts, comments, and other non-related studies; (2) Studies including other cases such as benign lung diseases; (3) Studies without necessary data for statistical analysis.

### Data extraction and quality assessment of included studies

Two reviewers independently extracted the data from all included researches and examined the results again carefully. If there were discrepancies, the controversial results were resolved by group discussion, and a final decision was made by a senior investigator. The following data were collected: first author, publication year, country, study design, sample size (RATS group and VATS group), mean age, sex, surgical techniques, tumor site, TNM stage, operative time, estimated blood loss, conversion, the number of dissected lymph nodes and stations, length of hospital stay, postoperative duration of drainage, postoperative complications, mortality, OS, DFS, recurrence rate, and cost. If the research reported medians and ranges, the means and standard deviations (SD) were calculated according to the method described by Hozo et al. [24]. The NOS was used to assess the quality of included studies. Scores range from 0 to 9 stars: studies with a score more than or equal to 7 were premeditated to be high-quality and included in the meta-analysis.

### Subgroup analysis

The uneven distribution of the surgical extension between the groups could affect the outcomes. Therefore, to eliminate the bias, a subgroup analysis of lung lobectomy or segmentectomy was conducted.

### Sensitivity analysis

Abraham et al. [25] proved that when comparing the short-term outcomes of surgery, the results obtained by combining high-quality non-randomized controlled trials were also convincing. To determine whether the pooled results are robust and reliable, a sensitivity analysis was performed by combining high-quality papers with more than 7 stars.

### Statistical analysis

All statistical analyses were performed using the Review Manager 5.3 software (Cochrane Collaboration, Oxford, UK). The dichotomous variables were assessed by using odds ratios (OR) with a 95% confidence interval (CI) and the continuous variables using weighted mean difference (WMD) with a 95% CI. The hazard ratio (HR) with its corresponding 95% CI was utilized to assess the survival data such as OS and DFS. The *I*^*2*^ statistics were used to evaluate the heterogeneity. *I*^*2*^ < 25, 25% ≤ *I*^*2*^ ≤ 50% and *I*^*2*^ > 50% were considered to be low, moderate, and high heterogeneity, respectively. If the test of heterogeneity was high (*I*^*2*^ > 50% or *P* < 0.05), a random-effect model was adopted. Otherwise, we used a fix effect model. The potential publication bias was evaluated by visually inspecting the funnel plots. *P* < 0.05 was regarded as statistically significant.

## Results

### Selected studies

**Fig. 1:**
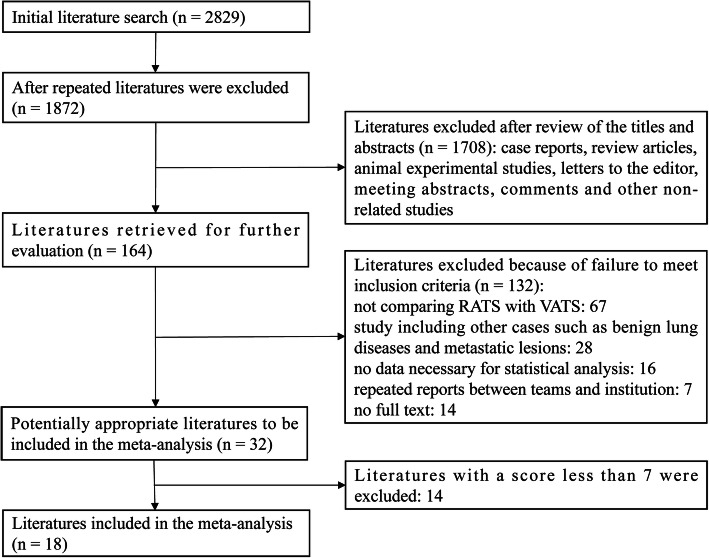
Flow chart of literature search strategies

A total of 2829 potential articles published before December 2020 were found from databases. After duplicates were removed, 1872 articles were initially evaluated by carefully reading the titles and abstracts, of which 1708 studies were excluded because they were review articles, case reports, animal experimental studies, letters, meeting abstracts, comments and other non-related studies. 164 potential articles were further assessed through reading the full texts, and finally, according to inclusion and exclusion criteria, a total of 18 retrospective studies were included in the final meta-analysis [26–43]. The flow chart of the screening strategies, which contains reasons for the exclusion of studies, is depicted in Fig. 1.

### Study characteristics and quality

A total of 18 studies with 11,247 patients, of whom 5114 patients were in the RATS group and 6133 in the VAST group, were involved in the analysis. Five included studies were published in Chinese [30, 31, 35–37], and 13 were published in English [26–29, 32–34, 38–43]. Among the 18 studies, 11 studies were from China [27, 30, 31, 33–37, 39, 41, 42], 6 from the USA [26, 28, 29, 32, 38, 43] and 1 from Japan [40]. The basic characteristics of the included studies are listed in Table 1. The quality assessment of the included studies according to the NOS is elucidated in Table 2. It showed that 9 of the 18 included studies had 7 stars [27, 28, 31–33, 35–37, 41], 4 had 8 stars [26, 30, 34, 42], and 5 had 9 stars [29, 38–40, 43].

**Table 1:** Characteristics of the included studies

Study	Year	Country	Design	Study Period	Group	Cases	Mean age	Sex (M/F)	Surgical techniques	Tumor Site (Right/Left)	TNM stage (I/II/III,IV)
Lee [26]	2015	USA	R	2009–2014	RATS	53	71.00	30/23	4 arms	99/59	NA
					VATS	158	72.00	56/102	2 ports	34/19	NA
Bao [27]	2016	China	R	2014–2015	RATS	69	58.60	26/43	NA	NA	65/1/3
					VATS	69	59.90	22/47	NA	NA	59/6/4
Oh [28]	2017	USA	R	2011–2015	RATS	2951	66.90	1369/1582	NA	NA	NA
					VATS	2951	66.70	1373/1578	NA	NA	NA
Yang [29]	2017	USA	R	2002–2012	RATS	172	68.00	74/98	3 arms or 4 arms	110/62	133/29/10
					VATS	141	67.50	53/88	3 ports	88/53	114/21/6
Liu [30]	2018	China	R	2012–2017	RATS	134	62.14	67/67	NA	81/53	134/0/0
					VATS	213	61.30	118/95	NA	117/96	213/0/0
Ma [31]	2019	China	R	2016–2017	RATS	37	61.14	26/11	NA	23/14	NA
					VATS	43	58.36	25/18	NA	27/16	NA
Merritt [32]	2019	USA	R	2014–2018	RATS	114	64.82	46/68	4 arms	61/53	71/27/9
					VATS	114	62.52	49/65	3 ports	68/46	79/22/9
Li JT [33]	2019	China	R	2013–2016	RATS	230	55.60	76/154	NA	152/78	230/0/0
					VATS	230	56.00	80/150	NA	162/68	230/0/0
Li [34]	2019	China	R	2014–2017	RATS	36	57.20	17/19	NA	23/13	0/16/20
					VATS	85	59.70	38/47	NA	51/34	0/36/49
Tong [35]	2020	China	R	2016–2018	RATS	33	61.97	19/14	2 arms	0/33	24/6/3
					VATS	41	62.24	27/14	3 ports	0/41	28/8/5
Huang [36]	2020	China	R	2019–2019	RATS	23	53.98	10/13	3 arms	15/8	19/2/2
					VATS	30	56.39	17/13	3 ports	19/11	22/6/2
Tian [37]	2020	China	R	2015–2018	RATS	283	57.90	128/155	3 arms	283/0	226/45/12
					VATS	296	58.76	118/178	4 ports	296/0	240/35/21
Veluswamy [38]	2020	USA	R	2008–2013	RATS	338	73.00	148/190	NA	NA	241/62/35
					VATS	1230	72.00	542/688	NA	NA	918/214/98
Zhou [39]	2020	China	R	2011–2018	RATS	50	54.70	15/35	4 arms	27/23	50/0/0
					VATS	80	57.70	26/54	4 ports	31/49	80/0/0
Haruki [40]	2020	Japan	R	2011–2018	RATS	49	70.00	21/28	4 arms	32/17	43/3/3
					VATS	49	68.00	24/25	3 ports	26/23	42/1/6
Zhang [41]	2020	China	R	2015–2019	RATS	257	53.53	84/173	4 arms	127/130	NA
					VATS	257	52.21	89/168	NA	124/133	NA
Qiu [42]	2020	China	R	2012–2017	RATS	40	61.40	36/4	3 arms	24/16	NA
					VATS	38	61.70	34/4	2 ports or 3 ports	17/21	NA
Kneuertz [43]	2020	USA	R	2012–2017	RATS	245	65.30	47/53	4 arms	61/40	86/10/5
					VATS	118	64.60	45/55	4 ports	58/42	90/6/4

*R* retrospective, *RATS* robot-assisted thoracic surgery, *VATS* video-assisted thoracic surgery, *M* male, *F* female, *NA* not available

**Table 2:** Assessment of the quality of the studies based on the NOS

Study	Selection (Out of 4)				Comparability (Out of 2)	Outcomes (Out of 3)			Total (Out of 9)
	(1)	(2)	(3)	(4)		(5)	(6)	(7)	
Lee [26]	*	*	*	*	*	*	*	*	8
Bao [27]	*	*	*	*	**	*			7
Oh [28]	*	*	*	*	**	*			7
Yang [29]	*	*	*	*	**	*	*	*	9
Liu [30]	*	*	*	*	*	*	*	*	8
Ma [31]	*	*	*	*	**	*			7
Merritt [32]	*	*	*	*	**	*			7
Li JT [33]	*	*	*	*	**	*			7
Li [34]	*	*	*	*	**	*	*		8
Tong [35]	*	*	*	*	**	*			7
Huang [36]	*	*	*	*	**	*			7
Tian [37]	*	*	*	*	**	*			7
Veluswamy [38]	*	*	*	*	**	*	*	*	9
Zhou [39]	*	*	*	*	**	*	*	*	9
Haruki [40]	*	*	*	*	**	*	*	*	9
Zhang [41]	*	*	*	*	**	*			7
Qiu [42]	*	*	*	*	*	*	*	*	8
Kneuertz [43]	*	*	*	*	**	*	*	*	9

(1) Representativeness of the exposed cohort, (2) selection of the non-exposed cohort, (3) ascertainment of exposure, (4) demonstration that outcome of interest was not present at the start of the study, (5) assessment of outcome, (6) was follow-up long enough for outcomes to occur, (7) adequacy of follow-up of cohorts

### Short-term outcomes

**Fig. 2:**
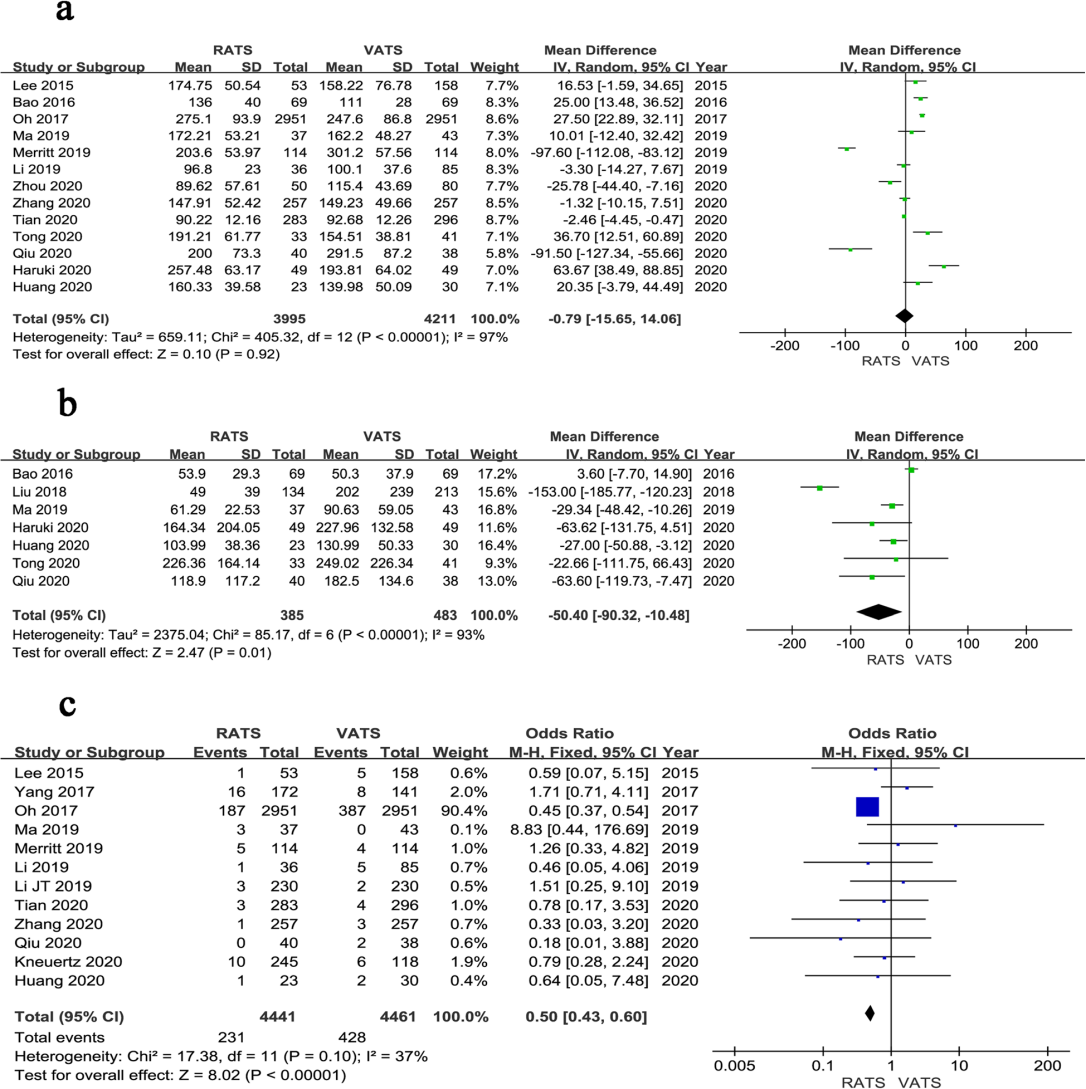
Forest plot of the meta-analysis for intraoperative parameters.**a** Operation time. **b** Estimated blood loss. **c** Conversion

**Fig. 3:**
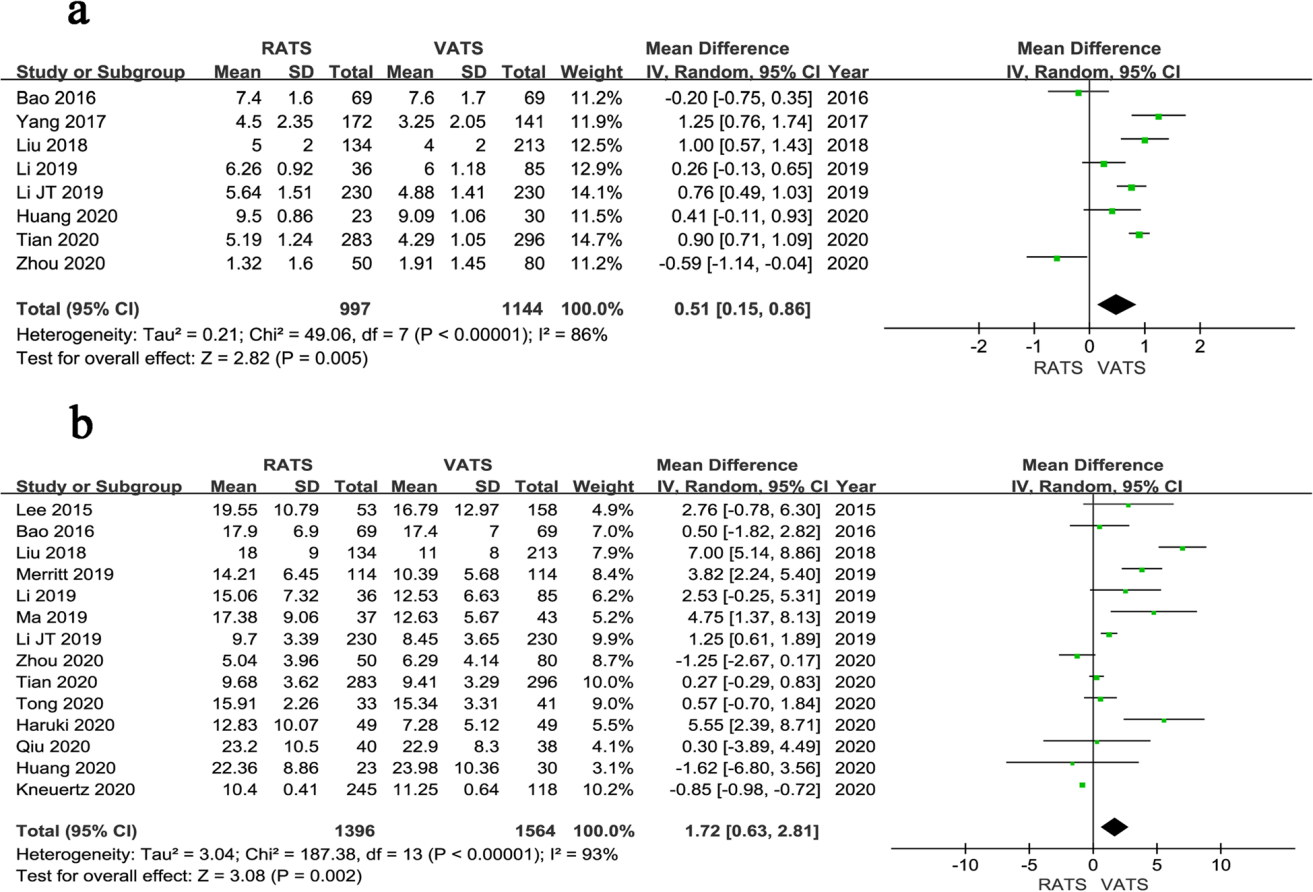
Forest plot of the meta-analysis for pathology details.**a** Number of dissected lymph node stations. **b** Number of dissected lymph nodes

**Fig. 4:**
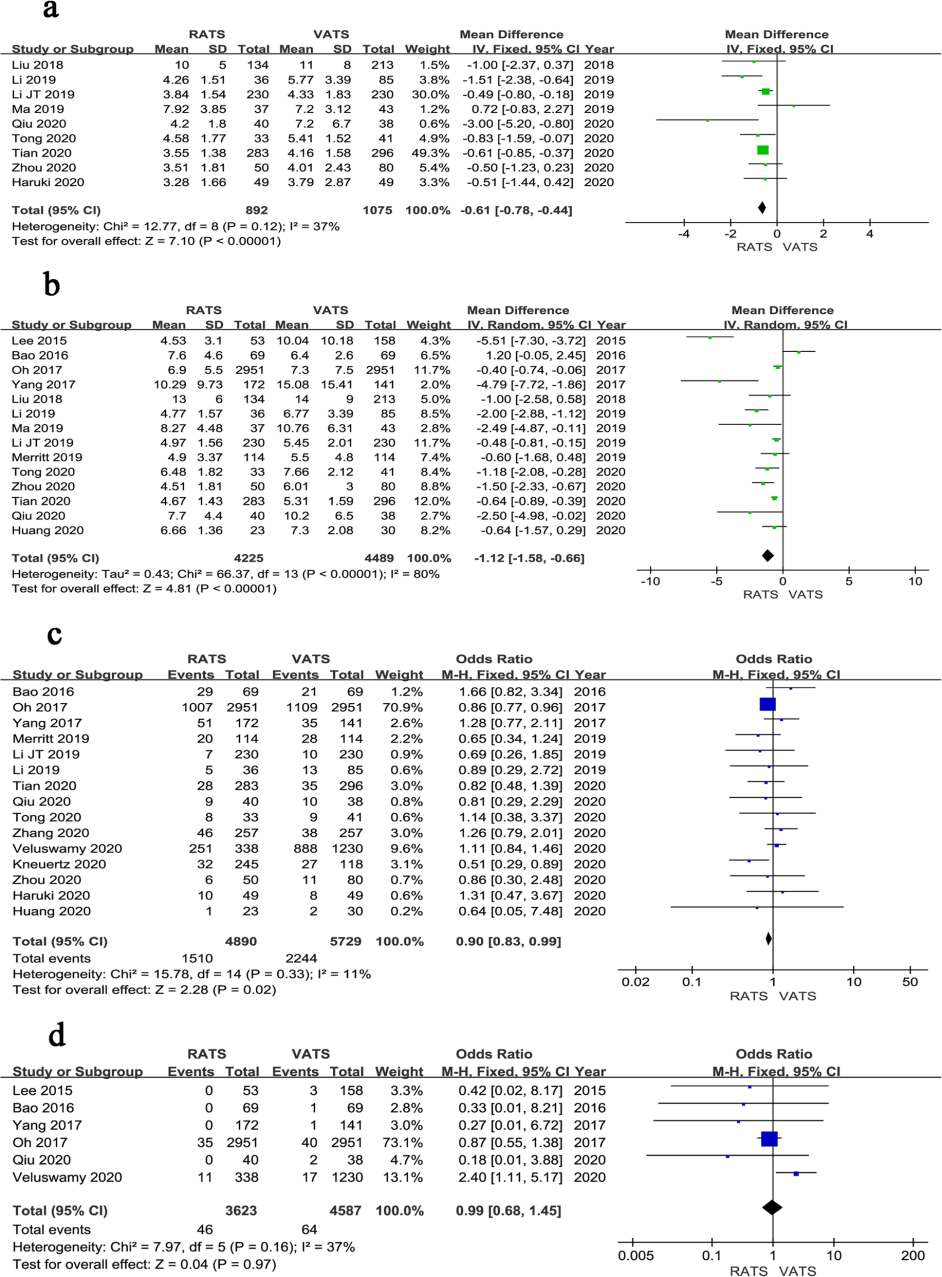
Forest plot of the meta-analysis for postoperative parameters.**a** Time of chest tube drainage. **b** Length of hospital stay. **c** Overall complications. **d** Mortality

All results of the meta-analysis for short-term outcomes are shown in Figs. 2, 3 and 4, which are summarized in Table 3. Thirteen studies reported the operative time. The pooled data based on 13 studies revealed no significant difference between the groups of RATS and VATS (WMD = − 0.79, 95% CI − 15.65 ~ 14.06, *P* = 0.920, *I*^*2*^ = 97%) (Fig. 2a). The EBL was reported in 7 studies. Pooled analysis of the data showed that the EBL was less in RATS than VATS (WMD = − 50.40, 95% CI − 90.32 ~ − 10.48, *P* = 0.010, *I*^*2*^ = 93%) (Fig. 2b). The data regarding the conversion cases were reported in 12 studies. Meta-analysis showed that the conversion rate of RATS group was lower than that of VATS group (OR = 0.50, 95% CI 0.43 ~ 0.60, *P* < 0.001, *I*^*2*^ = 37%) (Fig. 2c).

**Table 3:** Results of the meta-analysis

Outcomes	No. of studies	Sample size		Heterogeneity		Overall effect size	95% CI of overall effect	*P* Value
		RATS	VATS	I^2^(%)	*P* Value			
Operation time (min)	13	3995	4211	97	< 0.001	WMD = -0.79	-15.65 ~ 14.06	0.920
Estimated blood loss (mL)	7	385	483	93	< 0.001	WMD = -50.40	-90.32 ~ −10.48	0.010
Conversion	12	4441	4461	37	0.100	OR = 0.50	0.43 ~ 0.60	< 0.001
Dissected lymph node stations	8	997	1144	86	< 0.001	WMD = 0.51	0.15 ~ 0.86	0.005
Dissected lymph nodes	14	1396	1564	93	< 0.001	WMD = 1.72	0.63 ~ 2.81	0.002
Time of chest tube drainage (days)	9	892	1075	37	0.120	WMD = -0.61	-0.78 ~ −0.44	< 0.001
Length of hospital stay (days)	14	4225	4489	80	< 0.001	WMD = -1.12	-1.58 ~ − 0.66	< 0.001
Overall complications	15	4890	5729	11	0.330	OR = 0.90	0.83 ~ 0.99	0.020
Mortality	6	3623	4587	37	0.160	OR = 0.99	0.68 ~ 1.45	0.970
Overall survival	4	659	1471	28	0.240	HR = 1.02	0.82 ~ 1.26	0.880
Disease-free survival	2	76	123	0	0.490	HR = 1.03	0.66 ~ 1.61	0.890
Recurrence rate	6	605	631	0	0.490	OR = 0.51	0.36 ~ 0.72	< 0.001
Cost (USD)	4	615	636	0	0.550	WMD = 3909.87	3706.90 ~ 4112.84	< 0.001

The pooled results of 8 studies showed that the number of dissected lymph nodes stations was more in RATS than VATS (WMD = 0.51, 95% CI 0.15 ~ 0.86, *P* = 0.005) (Fig. 3a), with a high heterogeneity (*I*^*2*^ = 86%), which made a random effect model adopted. Pooled analysis of 14 studies showed that the number of dissected lymph nodes of RATS group was more than that of VATS group (WMD = 1.72, 95% CI 0.63 ~ 2.81, *P* = 0.002, *I*^*2*^ = 93%) (Fig. 3b).

The time of chest tube drainage was reported in 9 studies. Pooled analysis of the data showed that the time of chest tube drainage was shorter in RATS than VATS (WMD = − 0.61, 95% CI − 0.78 ~ − 0.44, *P* < 0.001, *I*^*2*^ = 37%) (Fig. 4a). The result of pooled analysis of 14 studies revealed that the length of hospital stay was shorter in RATS than that of VATS (WMD = − 1.12, 95% CI − 1.58 ~ − 0.66, *P* < 0.001, *I*^*2*^ = 80%) (Fig. 4b). Fifteen studies including a total of 10,619 patients presented the overall postoperative complication. Pooled analysis showed that the rate of overall postoperative complication was lower in RATS than VATS (OR = 0.90, 95% CI 0.83 ~ 0.99, *P* = 0.020, *I*^*2*^ = 11%) (Fig. 4c). Moreover, six studies reported the postoperative mortality and no significant difference was identified between the two groups (OR = 0.99, 95% CI 0.68 ~ 1.45, *P* = 0.970, *I*^*2*^ = 37%) (Fig. 4d).

### Long-term outcomes

**Fig. 5:**
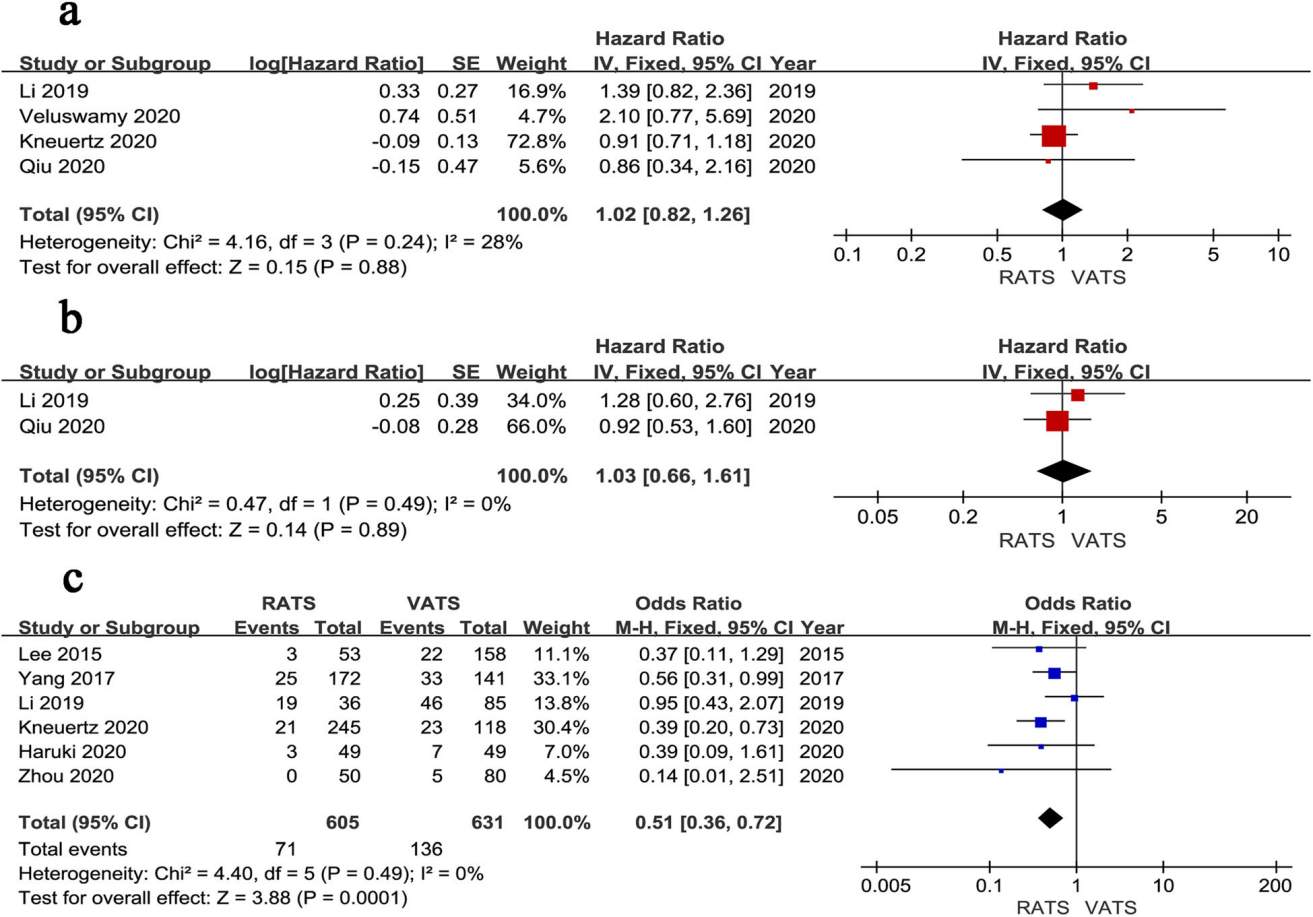
Forest plot of the meta-analysis for survival outcomes.**a** Overall survival.**b** Disease-free survival.**c** Recurrence rate

All results of meta-analysis for long-term outcomes are shown in Fig. 5, which are outlined in Table 3. Four studies described the OS and no significant difference could be found in OS between the two techniques (HR = 1.02, 95% CI 0.82 ~ 1.26, *P* = 0.880, *I*^*2*^ = 28%) (Fig. 5a). The DFS outcomes were recorded in 2 studies. The pooled results indicated that the DFS outcomes were similar between the two groups (HR = 1.03, 95% CI 0.66 ~ 1.61, *P* = 0.890), with no significant heterogeneity (*I*^*2*^ = 0%) (Fig. 5b). Cancer recurrence was reported in six studies and the pooled data indicated that the recurrence rate of RATS was lower than that of VATS (OR = 0.51, 95% CI 0.36 ~ 0.72, *P* < 0.001, *I*^*2*^ = 0%) (Fig. 5c).

### Total cost

**Fig. 6:**
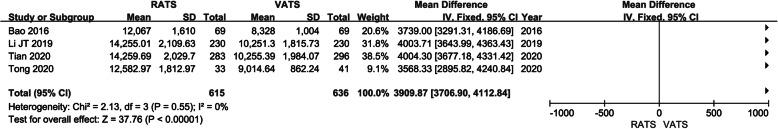
Forest plot of the meta-analysis for total cost

Only four studies recorded their total cost and they all reported a higher cost for RATS than VATS. The meta-analysis demonstrated that the total cost of RATS groups was significantly higher than VATS groups (WMD = 3909.87 USD, 95% CI 3706.90 ~ 4112.84, *P <* 0.001, *I*^*2*^ = 0%) (Fig. 6).

### Subgroup analysis

For the subgroup analysis of lung lobectomy, the RATS group was still associated with lower conversion rate, more harvested lymph nodes and stations, shorter duration of postoperative chest tube drainage and hospital stay, lower overall complication rate, and lower recurrence rate (*P* < 0.05), and had similar operation time compared with VATS (*P* > 0.05), which was highly consistent with the results of pooled analysis. As for lung segmentectomy, although there was no significant difference in conversion rate, harvested lymph nodes, duration of postoperative chest tube drainage, overall complication rate and recurrence rate (*P* > 0.05), the trend of RATS over VATS remained unchanged. The subgroup analysis results of surgical extension are summarized in Table 4.

**Table 4:** Results of the subgroup analysis of lung lobectomy or segmentectomy

Outcomes	No. of studies	Sample size		Heterogeneity		Overall effect size	95% CI of overall effect	*P* Value
		RATS	VATS	I^2^(%)	*P* Value			
Operation time (min)								
Lobectomy	10	3619	3805	98	<0.001	WMD = -1.13	−19.88 ~ 17.62	0.910
Segmentectomy	2	307	337	82	0.020	WMD = -12.12	−35.93 ~ 11.69	0.320
Conversion								
Lobectomy	11	4184	4204	42	0.070	OR = 0.51	0.43 ~ 0.60	<0.001
Segmentectomy	1	257	257	NA	NA	OR = 0.33	0.03 ~ 3.20	0.340
Dissected lymph node stations								
Lobectomy	6	878	995	66	0.010	WMD = 0.77	0.52 ~ 1.01	<0.001
Segmentectomy	1	50	80	NA	NA	WMD = -0.59	−1.14 ~ −0.04	0.030
Dissected lymph nodes								
Lobectomy	12	1277	1415	94	<0.001	WMD = 2.14	0.91 ~ 3.37	<0.001
Segmentectomy	1	50	80	NA	NA	WMD = -1.25	−2.67 ~ 0.17	0.090
Time of chest tube drainage (days)								
Lobectomy	8	842	995	61	0.010	WMD = -0.62	−1.01 ~ −0.23	0.002
Segmentectomy	1	50	80	NA	NA	WMD = -0.50	−1.23 ~ 0.23	0.180
Length of hospital stay (days)								
Lobectomy	12	4106	4340	80	<0.001	WMD = -1.23	−1.70 ~ −0.75	<0.001
Segmentectomy	1	50	80	NA	NA	WMD = -1.50	−2.33 ~ −0.67	<0.001
Overall complications								
Lobectomy	11	4176	4093	0	0.670	OR = 0.86	0.78 ~ 0.94	0.002
Segmentectomy	2	307	337	0	0.520	OR = 1.18	0.77 ~ 1.81	0.450
Recurrence rate								
Lobectomy	5	555	551	0	0.47	OR = 0.53	0.37 ~ 0.74	<0.001
Segmentectomy	1	50	80	NA	NA	OR = 0.14	0.01 ~ 2.51	0.180

*NA* not available

### Sensitivity analysis

**Fig. 7:**
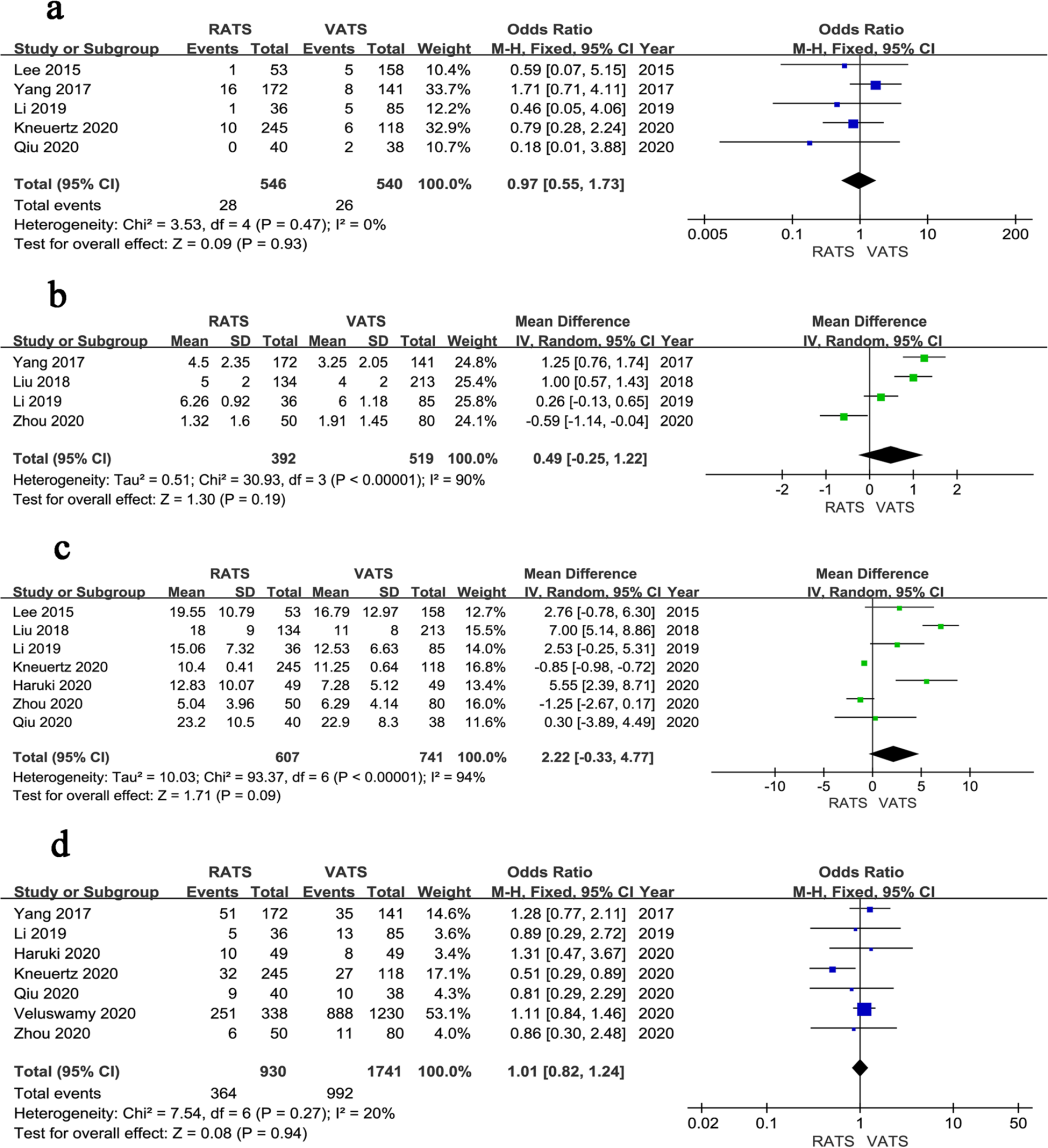
Forest plot of the sensitivity analysis. **a** Conversion. **b** Number of dissected lymph node stations. **c** Number of dissected lymph nodes. **d** Overall complications

We performed a sensitivity analysis for high-quality studies with more than 7 stars. The results of the sensitivity analysis showed that there were no significant difference between RATS and VATS on conversion rate (OR = 0.97, 95% CI 0.55 ~ 1.73, *P* = 0.930, *I*^*2*^ = 0%) (Fig. 7a), the number of dissected lymph node stations (WMD = 0.49, 95% CI -0.25 ~ 1.22, *P* = 0.190, *I*^*2*^ = 90%) (Fig. 7b), the number of dissected lymph nodes (WMD = 2.22, 95% CI -0.33 ~ 4.77, *P* = 0.090, *I*^*2*^ = 94%) (Fig. 7c), and the rate of overall postoperative complication (OR = 1.01, 95% CI 0.82 ~ 1.24, *P* = 0.940, *I*^*2*^ = 20%) (Fig. 7d), but with only a marginal difference compared to the results of pooled analysis. In general, the tendency was not changed. Compared with the overall outcomes, no significant changes were found in the remaining results. The results are shown in the Supplementary.

### Publication of bias

**Fig. 8:**
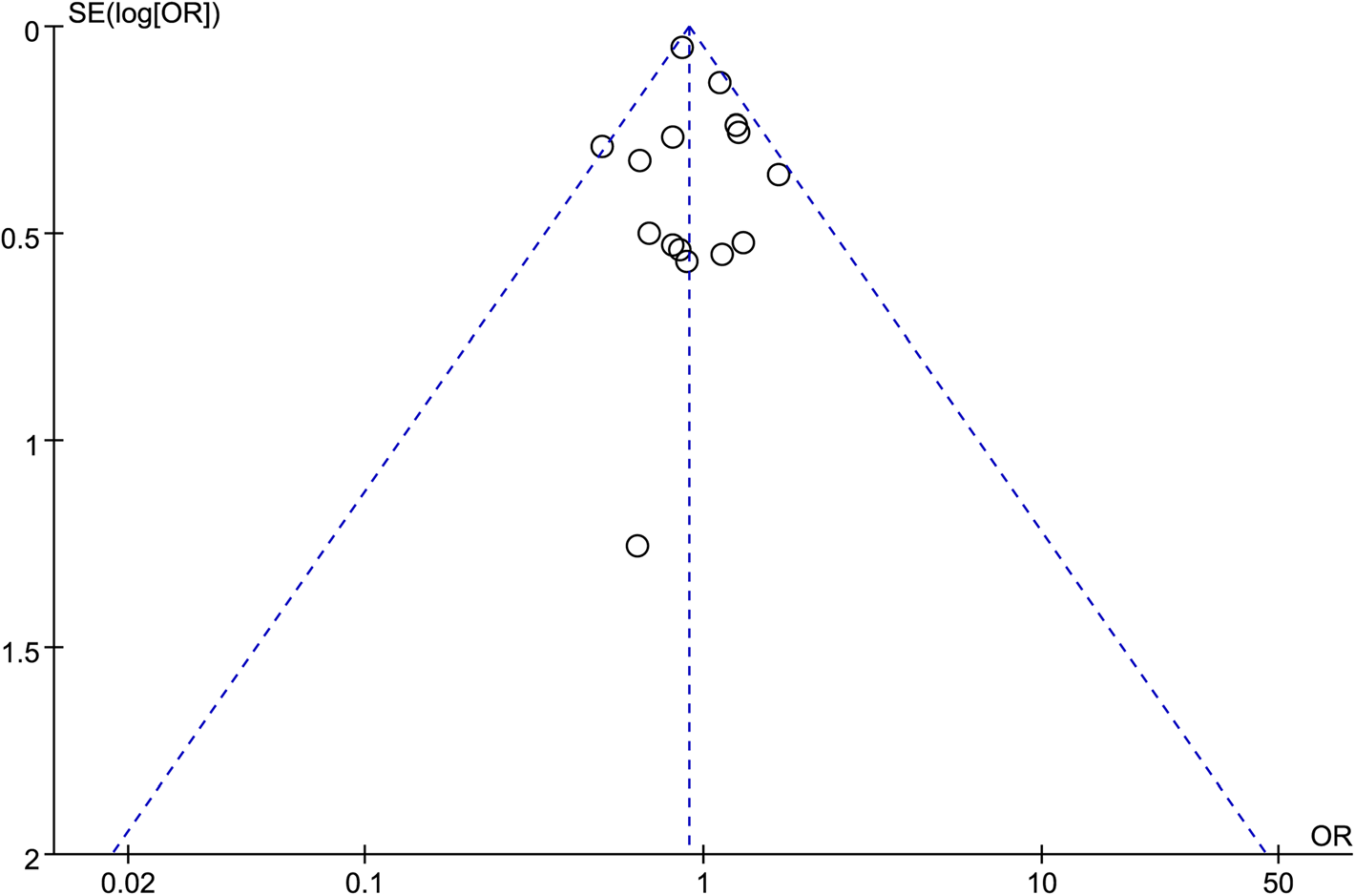
Funnel plot of the overall postoperative complications

A funnel plot of the overall complication was used to assess publication bias. The bilaterally symmetrical funnel plot of overall complication showed that no obvious evidence of publication bias was observed (Fig. 8).

## Discussion

Radical resection with lymphadenectomy is regarded as a gold standard surgical approach of treatment for patients with NSCLC at an early stage [4, 37]. As numerous researchers have proven its safety and effectiveness, MIS used to treat early-stage NSCLC has rapidly spread during the past decade. Minimally invasive VATS has been suggested for resection of NSCLC as a consequence of the improved short outcomes, such as shorter length of chest tube duration, lower rates of complications, and reduced length of stay, while maintaining an equivalent long-term survival rate compared to open thoracotomy [44]. Many studies have compared the safety and short-term or long-term efficacy of VATS with open thoracotomy, but studies on RATS have not been sufficient to prove its benefits. Therefore, we included 18 studies and conducted a meta-analysis to explore and compare the clinical efficacy of RATS and VATS.

Regarding the operative time, the result of meta-analysis revealed that the operative time was similar between the two groups, with no statistical difference, which was consistent with the results reported by Liang et al. [45]. However, most previous studies reported a longer operative time for RATS compared to VATS, which was contrary to our results [26, 46–48]. The main reason might come from the difference of the experience of surgeons. Previous researchers just reported their initial attempts to RATS for lung cancer and the experience and knowledge of RATS for surgeons were inadequate at the beginning of the learning curve, which might contribute to longer operative time. In our included studies, some surgeons had proficient operation experience in thoracoscopic lung cancer resection, which made the learning curve for robot-assisted lung cancer resection shorter and shortened the operative time significantly. Zhou et al. [39] described that the RATS group showed a sharper concurrent reduction in operation time than that for VATS after the surgeons performing RATS had obtained more than 10 years of advanced experience in performing VATS procedures. Merritt et al. [32] reported that the mean operative time for the final 20 RATS procedures was significantly shorter than that of the first 20 RATS procedures. Therefore, with the increased experience of RATS, operative time for RATS would be comparable to VATS.

Intraoperative blood loss mainly occurs during lymph node dissection, which is caused by vascular damage. Analysis of the pooled data showed that the blood loss was lower in RATS than VATS. The reason may be that RATS provides a three-dimensional magnified vision and more flexible equipments, eliminates hand tremors and accurately reveals the complex anatomy around the mediastinum and hilar, which helps surgeons perform precise manipulation and better control bleeding in small blood vessels [31]. In terms of the conversion rate, the results of this meta-analysis suggested that the conversion rate of RATS group was lower than that of VATS, but this was not consistent with the result of sensitivity analysis. Although no significant difference could be found in the conversion rate between the two techniques according to the result of sensitivity analysis, the pooled result revealed that VATS group still had a higher conversion rate than RATS group. The main reason may be that the robotic system has flexible operating instruments and has certain advantages in dealing with severe lymph node calcification and extensive chest adhesions, which are often the cause of conversion to open surgery [37].

Lymph node dissection is an important factor affecting the prognosis of NSCLC. Previous studies showed that there was no significant difference between RATS and VATS in the number of dissected lymph nodes and stations [33, 49]. Meanwhile, there were also studies that revealed that RATS could achieve more lymph node dissections than VATS [50, 51]. The results of this study were also more inclined to the latter. The mediastinum and hilar lymph nodes are relatively deep, and the adjacent anatomy is complicated. Therefore, the dissection of the mediastinum and hilar lymph nodes has always been a major test for the technique of the surgeons and the flexibility of surgical instruments [52]. The robotic surgery system has an internal articulated EndoWrist with 7° of freedom, which makes it more accurate than VATS in the separation of deep tissues and the removal of lymph nodes in complex anatomical locations. Postoperative pathological lymph node upstaging can better reflect the thoroughness of lymph node dissection. Wilson et al. [53] and Kneuertz et al. [54] reported a rate of lymph node upstaging for robotic resection that appeared to be superior to VATS, which indicated that the lymph node dissection of RATS was more thorough. After we conducted a sensitivity analysis, the results showed that the average number of dissected lymph nodes and stations in the RATS group was more than that in the VATS group, but the difference between the two groups was not statistically significant, which may be related to the small sample size of the included patients, and the statistical results were difficult to reflect the difference between the two groups. Further prospective studies are needed in order to confirm these results. Nevertheless, these could at least show that robotic surgery could achieve similar results with VATS.

In this study, the duration of postoperative chest tube drainage and hospital stay of patients in the RATS group were shorter than those in the VATS group, which was similar to the results of other studies [35, 55]. The possible reason is that the robotic surgery system has many minimally invasive advantages, which make the operation more delicate, more thorough hemostasis, less irritation to surrounding tissues such as pleura, resulting in less pleural effusion and saving the duration of postoperative drainage. In addition, patients recovered faster after surgery, which also shortened the postoperative hospital stay to a certain extent [56].

The incidence of postoperative complications is an important indicator for evaluating the short-term outcomes. The pooled result of the meta-analysis indicated that the postoperative complication rate in the group of RATS was less than in VATS group. It might be related to the continuous advancement of robotic surgery system and the improvement of the proficiency of surgeons in its operation, which made robotic surgery less harmful to patients and fewer postoperative complications [57]. However, the result of sensitivity analysis showed that there was no significant difference in the postoperative complication rate between the two groups. Perhaps it is related to the surgeon’s familiarity with the instrument, experience, and assistant compliance, which may affect the surgical results, such as postoperative complications. Therefore, this result should be interpreted with caution. Further prospective randomized controlled studies are needed in order to confirm the advantage. In terms of the mortality, analysis of the pooled data indicated that the mortality of the RATS group was similar to that of the VATS group, which was inconsistent with the result reported in a previous study that the mortality of RATS was significantly reduced compared with VATS [58]. It might be related to the highly selected patients at the beginning of this surgical technique. Sensitivity analysis also showed that there was no difference in mortality between the two techniques, which suggested that our result was robust and reliable. According to these results, we believe that RATS is safe and acceptable.

Because lung cancer is a malignant tumor, the long-term survival outcomes of patients with NSCLC requires special attention from the surgeons. As far as we know, our study analyzed the long-term survival outcomes of RATS and VATS for the first time, which was not analyzed in any previous meta-analysis. In our study, OS, a major oncologic outcome, was similar between the two groups. Pooled analysis showed no significant difference between the RATS and VATS groups in DFS, without significant heterogeneity. Regarding the recurrence rate, meta-analysis showed that it was significantly lower in RATS than VATS. According to the description of previous studies, tumor size, resection margin, lymph node dissection and time of follow-up were considered to be some of the factors influencing the postoperative recurrence of patients with NSCLC [59, 60]. One of the included studies reported that no local recurrence or distant recurrence appeared in the RATS group within the median 38-month follow-up time, while the local recurrence and distant recurrence rate in the VATS group was 2.5 and 3.75% respectively during a median follow-up time of 85 months [39]. Moreover, Li et al. [34] reported that the recurrence rate was 52.8% in the RATS group and 54.1% in the VATS group during a median follow-up length of 33.9 months. The above all indicated that the long-term oncologic outcomes of RATS might be slightly better than VATS and confirmed that RATS is a feasible and safe technique for the management of NSCLC. However, multi-center and large-scale clinical randomized controlled trials are needed to verify these results to provide more reliable evidence.

The high current cost of robotic thoracic surgery is worrying, which is mainly derived from the robotic system itself and may limit its uptake by thoracic surgeons. If RATS can reduce complications and shorten hospital stay, the higher costs of the robotic system would be partially offset. However, according to the results of Novellis et al. [22], although inpatient stay was shortest for robotic patients compared with VATS and open surgery, savings on this major item were insufficient to bring the cost of robotic surgery closer to those of VATS and open surgery. Therefore, it is essential for robotic operators to reduce operating time and robot consumables and inspect whether the potential advantages of the robotic approach justifies its high cost in the treatment of NSCLC in order for robotic surgery to be competitive. In addition, Novellis et al. [22] also reported that there was still a margin of profit for hospital even for patients treated with the robotic system, because it cost about 18% less than the current health service reimbursement.

To eliminate the bias, we conducted a subgroup analysis of lung lobectomy or segmentectomy. In our study, RATS was associated with lower conversion rate, more harvested lymph nodes, shorter duration of postoperative chest tube drainage, lower overall complication rate, and lower recurrence rate in the subgroup of lobectomy, contrasting with those in the subgroup of segmentectomy, which implied RATS to be superior to VATS when used in lobectomy. As for the subgroup of segmentectomy, compared with VATS, RATS had a slight advantage, with more harvested lymph node stations and shorter hospital stay. However, the sample size of the subgroup of segmentectomy was not large enough to be conclusive. Therefore, it needs to be confirmed by a multi-center randomized controlled study with a large sample.

The possible limitations of our study must be considered when applying these results in the clinic. First, all studies included for meta-analysis were retrospective observational studies and lacked high-quality randomized controlled trials, with a greater risk of potential selection and publication bias, which might influence the quality of meta-analysis. However, no significant publication bias was observed in our study. Second, we found that the operative time, blood loss, number of dissected lymph nodes and stations,and postoperative hospital stay had significant heterogeneity although the heterogeneity of other outcomes was not significant. Potential factors that could explain the heterogeneity included the different experience of surgeons and the shorter learning curve for the robotic group. Finally, the median, SD, HR, and standard error (SE) were not directly reported in some studies. The median and SD were calculated according to the methods described in previous studies, while HR and SE were extracted from the survival curves, which might result in a potential source of bias.

## Conclusion

In conclusion, our meta-analysis suggested that RATS is a feasible and safe technique that can achieve the same or even better surgical efficacy compared with VATS in terms of short-term and long-term outcomes. RATS makes up for the limitations of VATS in the treatment of NSCLC, which can make patients have less trauma and quicker recovery. However, large sample and high quality randomized clinical trials are still essential to compare RATS with VATS to better demonstrate the potential advantages and disadvantages of both of the minimally invasive approaches for NSCLC.

## Supplementary Information

**Additional file 1: Figure S1.** Sensitivity analysis result of estimated blood loss.

**Additional file 2: Figure S2.** Sensitivity analysis result of length of hospital stay.

**Additional file 3: Figure S3.** Sensitivity analysis result of mortality.

**Additional file 4: Figure S4.** Sensitivity analysis result of operative time.

**Additional file 5: Figure S5.** Sensitivity analysis result of postoperative duration of drainage.

**Additional file 6: Figure S6.** Subgroup analysis result of operation time. **Figure S7.** Subgroup analysis result of conversion. **Figure S8.** Subgroup analysis result of dissected lymph node stations. **Figure S9.** Subgroup analysis result of dissected lymph nodes. **Figure S10.** Subgroup analysis result of time of chest tube drainage. **Figure S11.** Subgroup analysis result of length of hospital stay. **Figure S12.** Subgroup analysis result of overall complications. **Figure S13.** Subgroup analysis result of recurrence rate.

## Data Availability

All data generated or analysed during this study are included in this published article and its supplementary information files.

## Abbreviations

CI: Confidence interval
CNKI: China national knowledge infrastructure
DFS: Disease-free survival
EBL: Estimated blood loss
F: Female
HR: Hazard ratios
M: Male
MIS: Minimally invasive surgery
NA: Not available
NOS: Newcastle-Ottawa Scale
NSCLC: Non-small cell lung cancer
OR: Odds ratios
OS: Overall survival
PRISMA: Preferred reporting items for systematic reviews and meta-analyses
R: Retrospective
RATS: Robot-assisted thoracic surgery
SD: Standard deviation
SE: Standard error
VATS: Video-assisted thoracic surgery
WMD: Weighted mean difference
