# Supplementary material for: Robot-assisted thoracic surgery versus video-assisted thoracic surgery for lung lobectomy or segmentectomy in patients with non-small cell lung cancer: a meta-analysis
Source: BMC Cancer. 2021 May 3;21:498. doi: 10.1186/s12885-021-08241-5 (PMC8094485; doi:10.1186/s12885-021-08241-5)
Supplement: Supplementary file 1 — Additional file 1: Figure S1. Sensitivity analysis result of estimated blood loss. [file 12885_2021_8241_MOESM1_ESM.docx]

**Robot-assisted thoracic surgery versus video-assisted thoracic surgery for lung lobectomy or segmentectomy in patients with non-small cell lung cancer: a meta-analysis**

Jianglei Ma^1*^ **·** Xiaoyao Li^1*^ **·** Shifu Zhao^1*^ **·** Jiawei Wang^1^ **·** Wujia Zhang^1^ **·** Guangyuan Sun^2＃^

^1^Student of the College of Basic Medical Sciences, Naval Medical University,Shanghai 200433, China

^2^Department of Thoracic Surgery, Changzheng Hospital, Naval Medical University, Shanghai 200003, China

**Institutional addresses:**

^1^No. 800 Xiangyin Road, Yangpu District, Shanghai 200433, China

^2^No. 415 Fengyang Road, Huangpu District, Shanghai 200003, China

***Co-first authors:** Jianglei Ma^1*^ & Xiaoyao Li^1*^ & Shifu Zhao^1*^

*****These authors contributed equally to this work.

**^＃^Corresponding author:** Guangyuan Sun^2＃^
**E-mail:** sunguangyuan@126.com


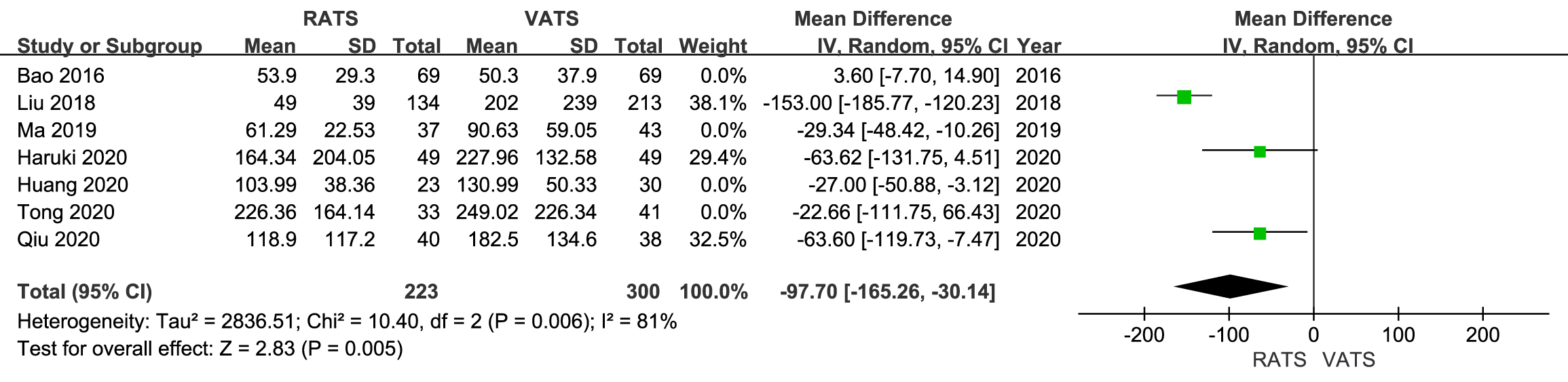


Figure S1. Sensitivity analysis result of estimated blood loss
