# Supplementary material for: Robot-assisted thoracic surgery versus video-assisted thoracic surgery for lung lobectomy or segmentectomy in patients with non-small cell lung cancer: a meta-analysis
Source: BMC Cancer. 2021 May 3;21:498. doi: 10.1186/s12885-021-08241-5 (PMC8094485; doi:10.1186/s12885-021-08241-5)
Supplement: Supplementary file 6 — Additional file 6: Figure S6. Subgroup analysis result of operation time. Figure S7. Subgroup analysis result of conversion. Figure S8. Subgroup analysis result of dissected lymph node stations. Figure S9. Subgroup analysis result of dissected lymph nodes. Figure S10. Subgroup analysis result of time of chest tube drainage. Figure S11. Subgroup analysis result of length of hospital stay. Figure S12. Subgroup analysis result of overall complications. Figure S13. Subgroup analysis result of recurrence rate. [file 12885_2021_8241_MOESM6_ESM.docx]

**Robot-assisted thoracic surgery versus video-assisted thoracic surgery for lung lobectomy or segmentectomy in patients with non-small cell lung cancer: a meta-analysis**

Jianglei Ma^1*^ **·** Xiaoyao Li^1*^ **·** Shifu Zhao^1*^ **·** Jiawei Wang^1^ **·** Wujia Zhang^1^ **·** Guangyuan Sun^2＃^

^1^Student of the College of Basic Medical Sciences, Naval Medical University,Shanghai 200433, China

^2^Department of Thoracic Surgery, Changzheng Hospital, Naval Medical University, Shanghai 200003, China

**Institutional addresses:**

^1^No. 800 Xiangyin Road, Yangpu District, Shanghai 200433, China

^2^No. 415 Fengyang Road, Huangpu District, Shanghai 200003, China

***Co-first authors:** Jianglei Ma^1*^ & Xiaoyao Li^1*^ & Shifu Zhao^1*^

*****These authors contributed equally to this work.

**^＃^Corresponding author:** Guangyuan Sun^2＃^
**E-mail:** [sunguangyuan@126.com](mailto:sunguangyuan@126.com)


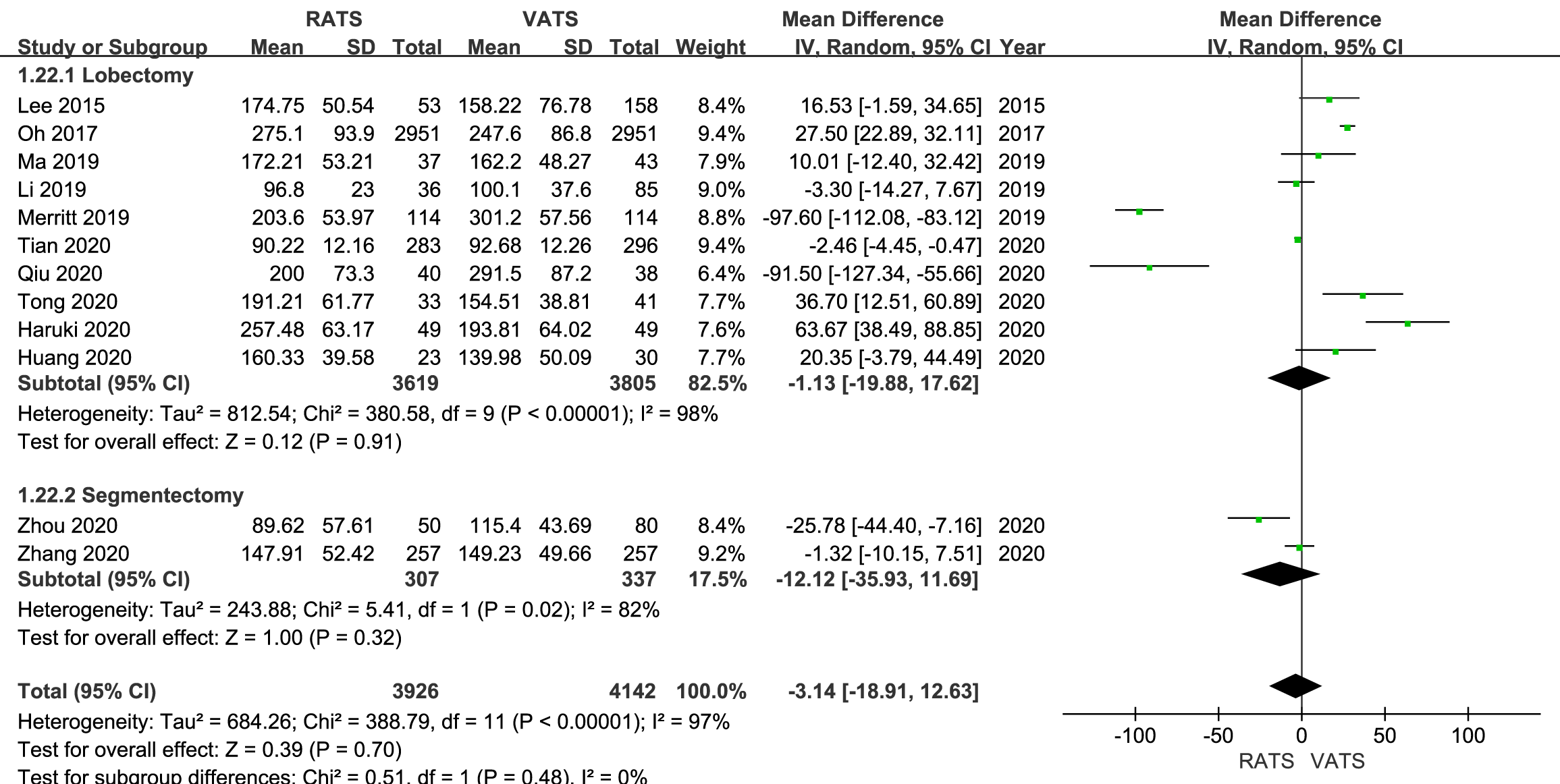


Figure S6. Subgroup analysis result of operation time


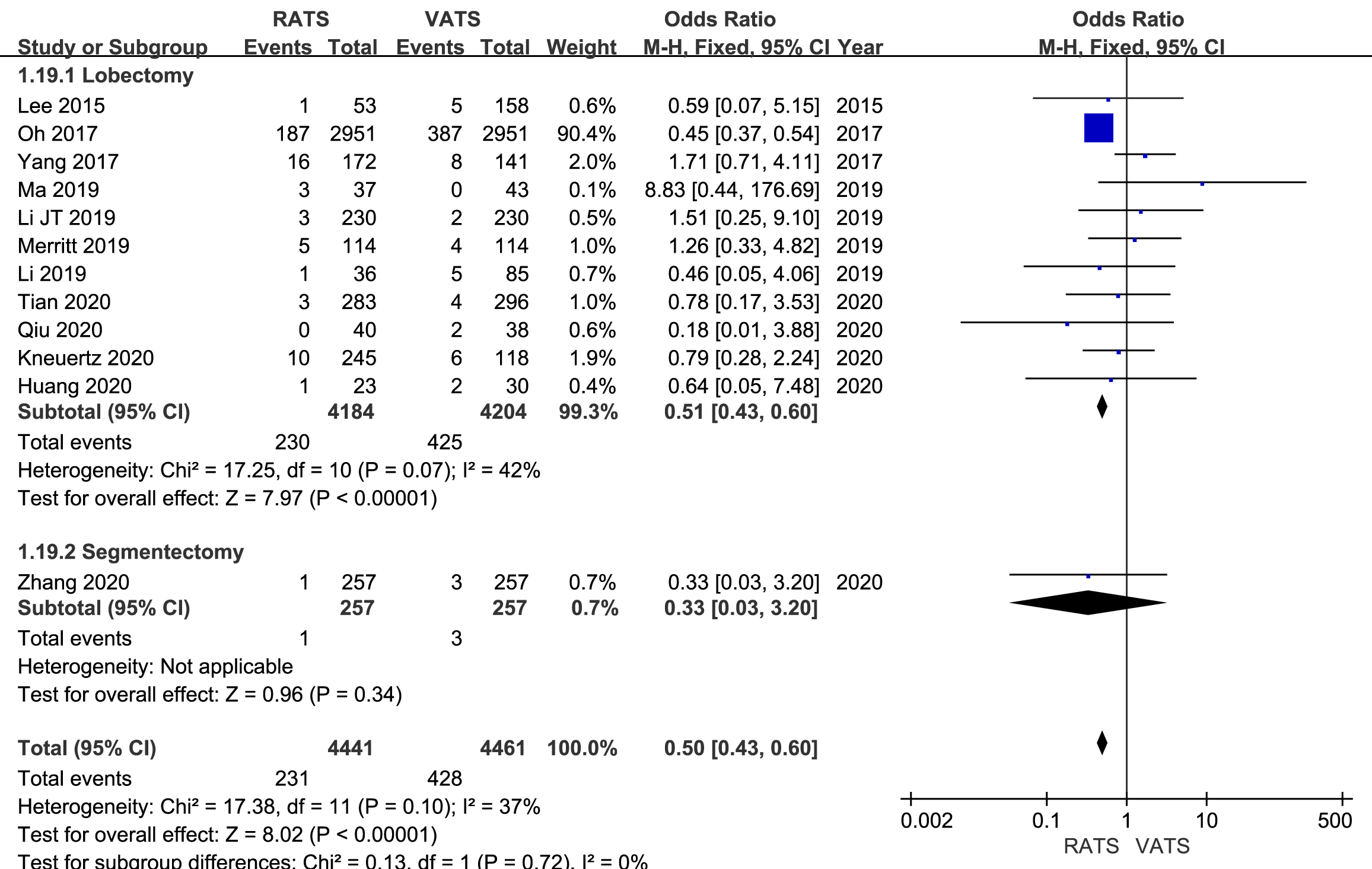


Figure S7. Subgroup analysis result of conversion


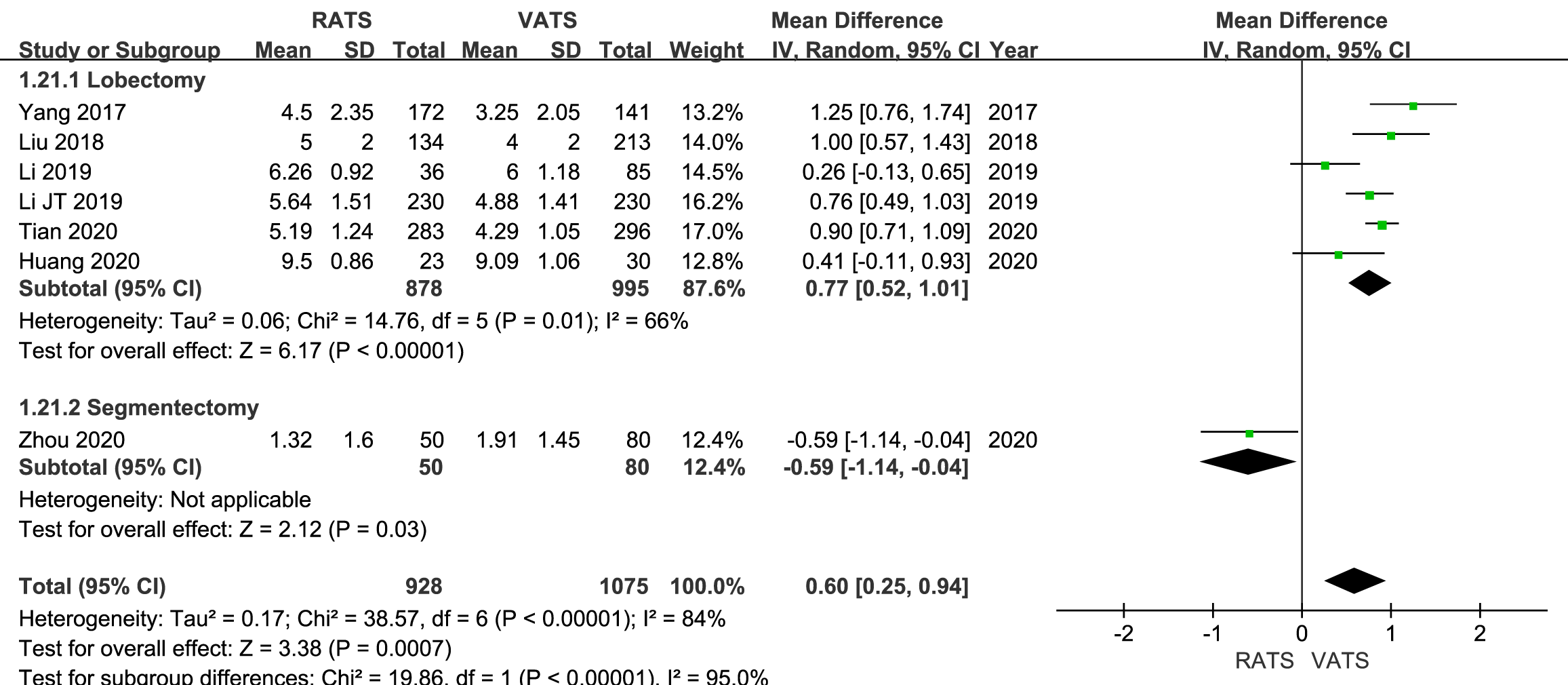


Figure S8. Subgroup analysis result of dissected lymph node stations


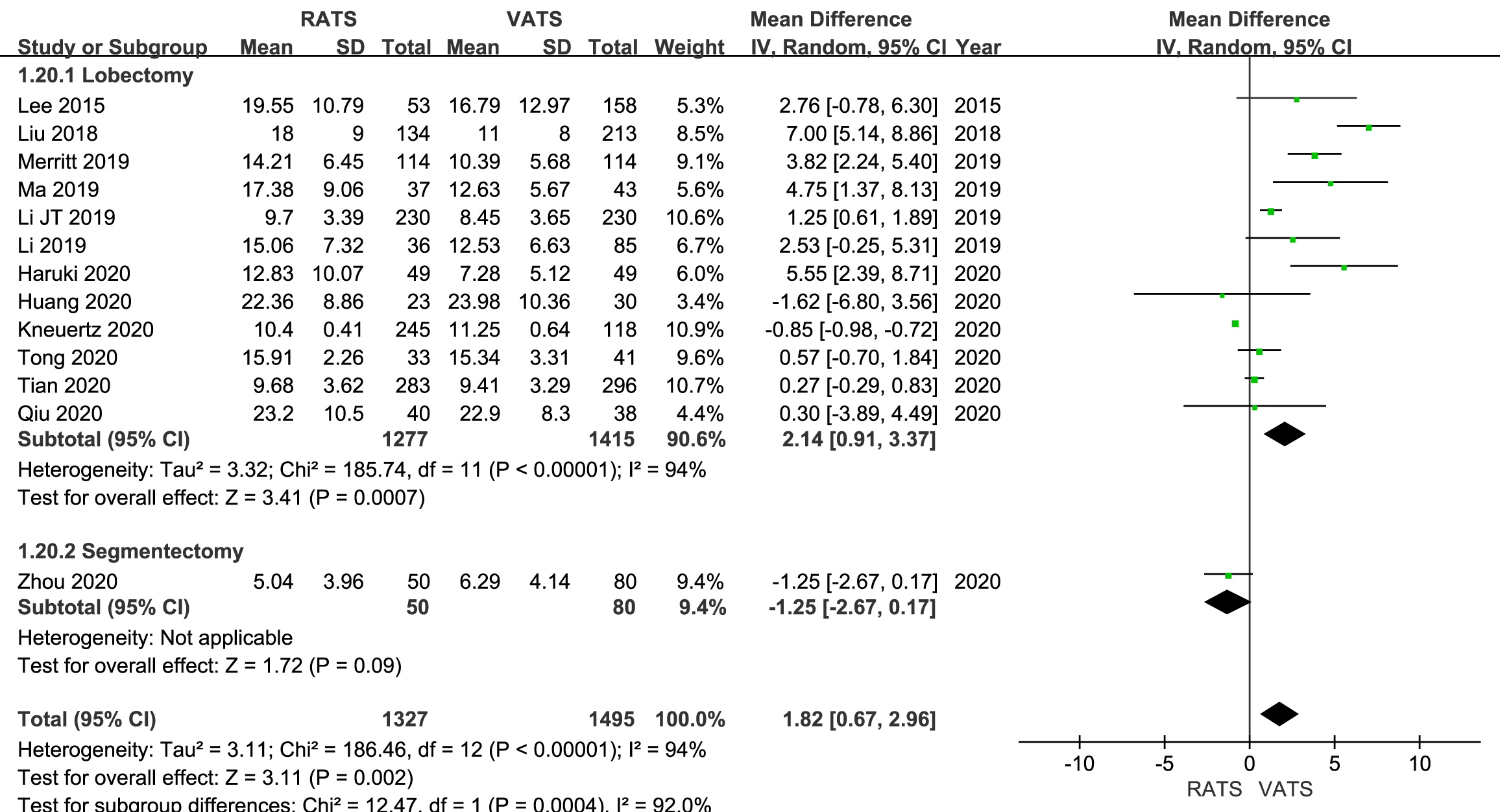


Figure S9. Subgroup analysis result of dissected lymph nodes


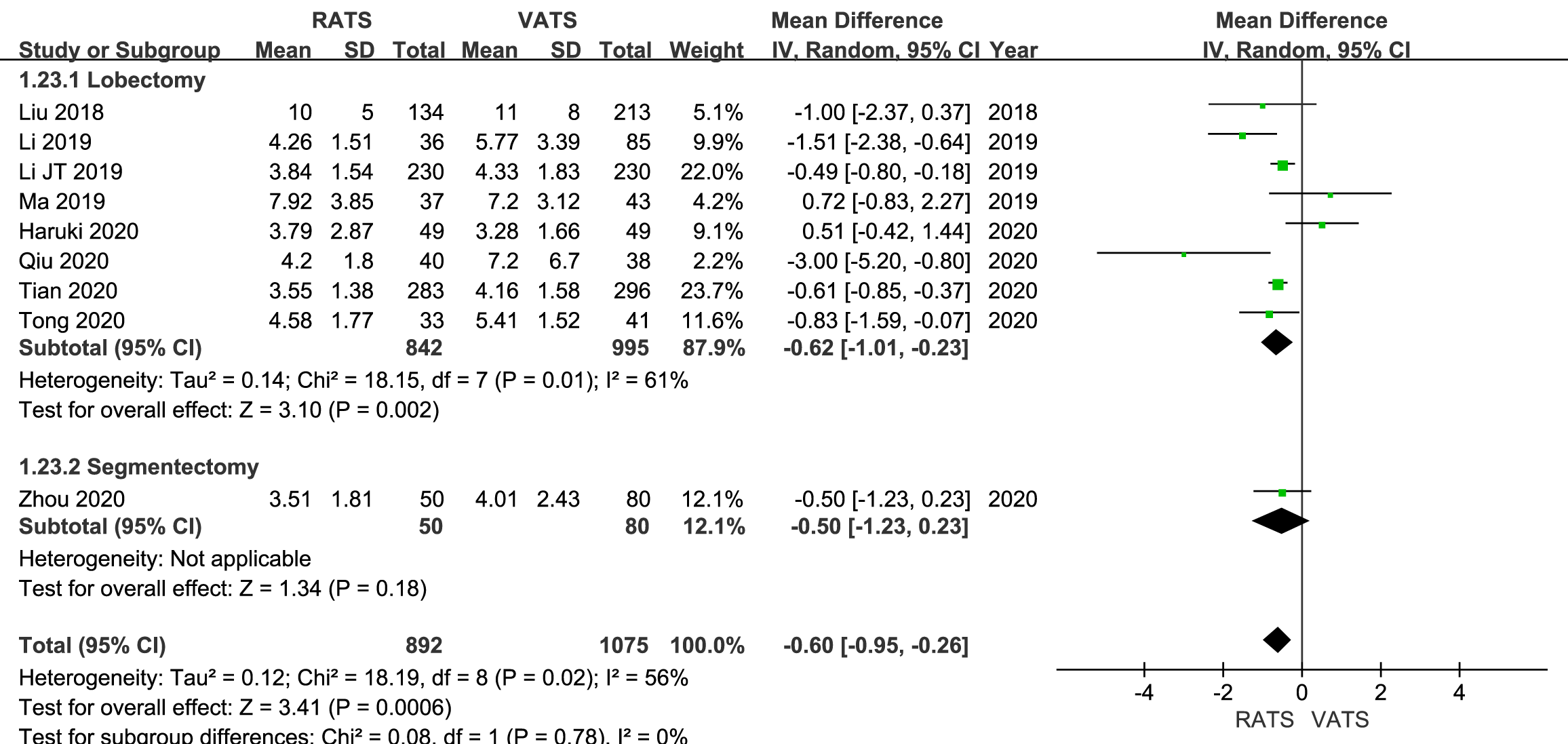


Figure S10. Subgroup analysis result of time of chest tube drainage


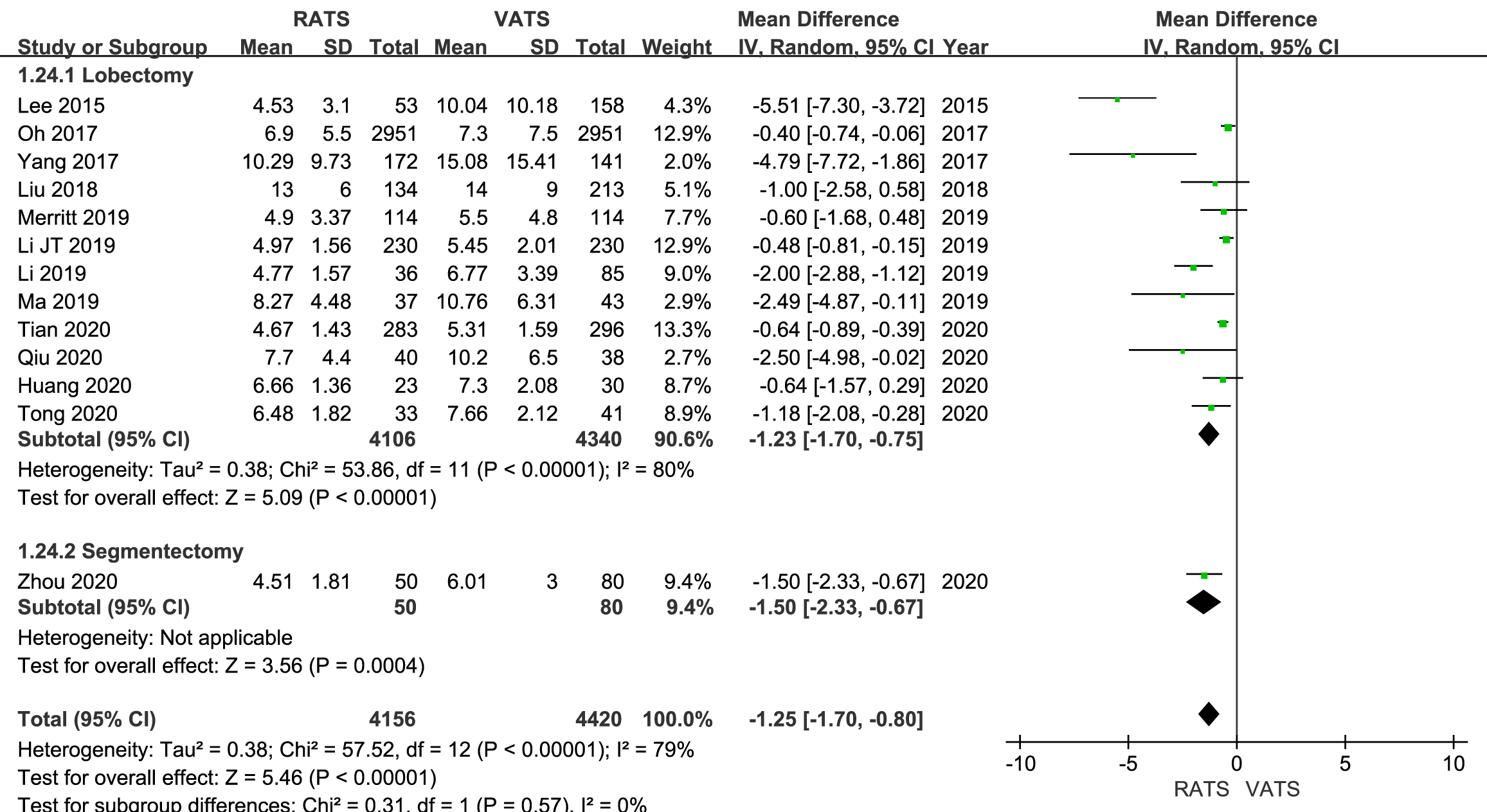


Figure S11. Subgroup analysis result of length of hospital stay


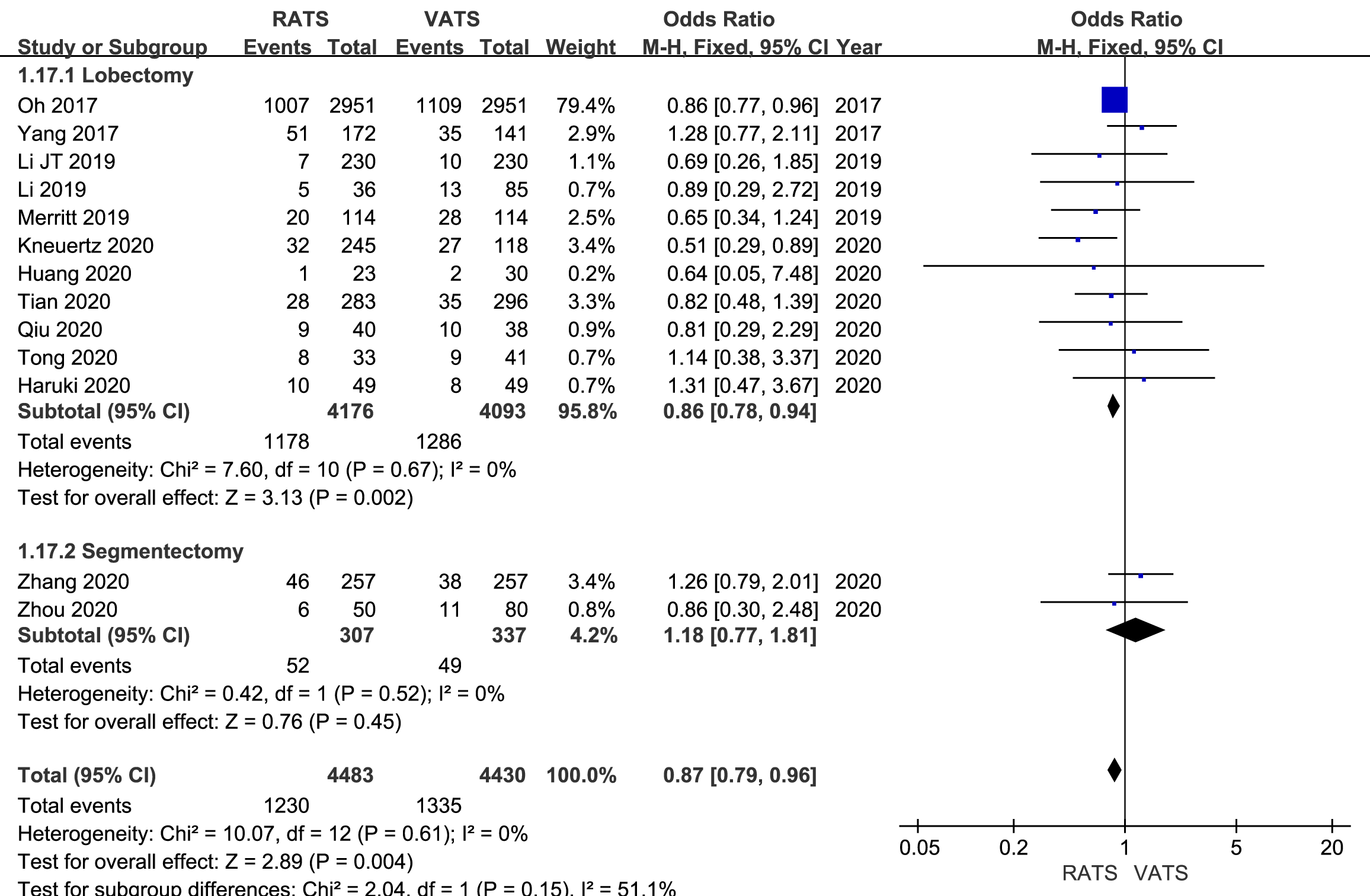


Figure S12. Subgroup analysis result of overall complications


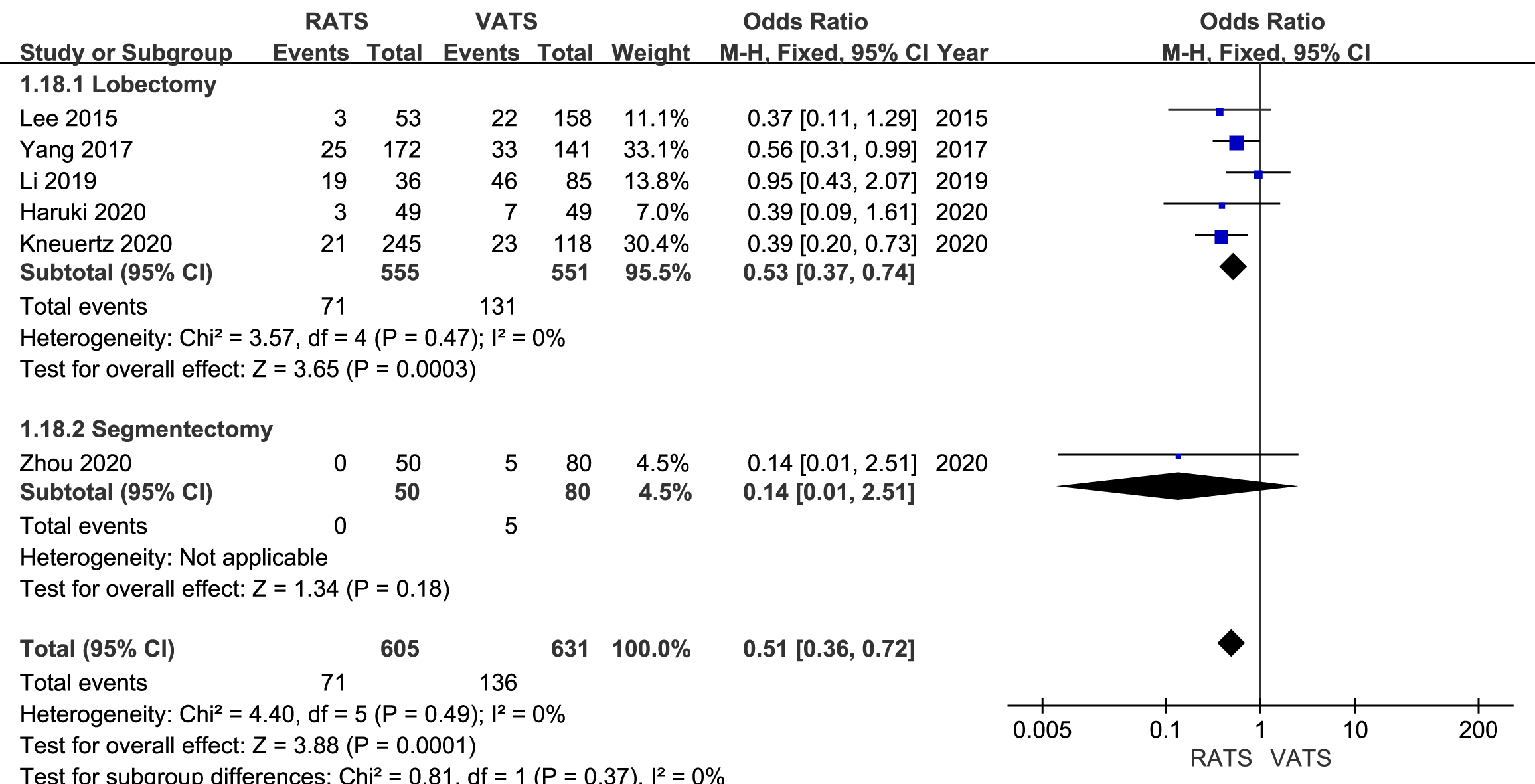


Figure S13. Subgroup analysis result of recurrence rate
